# Radiotherapy-induced alterations in tumor microenvironment: metabolism and immunity

**DOI:** 10.3389/fcell.2025.1568634

**Published:** 2025-04-28

**Authors:** Jinpeng Chen, Sheng Wang, Yue Ding, Duo Xu, Shiya Zheng

**Affiliations:** ^1^ Department of General Surgery, Zhongda Hospital Southeast University, Nanjing, Jiangsu, China; ^2^ Southeast University Medical School, Nanjing, Jiangsu, China; ^3^ Department of Radiation Oncology, Jiangsu Cancer Hospital, Jiangsu Institute of Cancer Research, Nanjing Medical University Affiliated Cancer Hospital, Nanjing, Jiangsu, China; ^4^ Department of Oncology, Tongji Hospital, Tongji Medical College, Huazhong University of Science and Technology, Wuhan, Hubei, China; ^5^ Department of Oncology, Southeast University, Zhongda Hospital Southeast University, Nanjing, Jiangsu, China

**Keywords:** tumor metabolism, tumor microenvironment, radiotherapy, immune modulation, metabolic reprogramming

## Abstract

Tumor metabolism plays a pivotal role in shaping immune responses within the tumor microenvironment influencing tumor progression, immune evasion, and the efficacy of cancer therapies. Radiotherapy has been shown to impact both tumor metabolism and immune modulation, often inducing immune activation through damage-associated molecular patterns and the STING pathway. In this study, we analyse the particular characteristics of the tumour metabolic microenvironment and its effect on the immune microenvironment. We also review the changes in the metabolic and immune microenvironment that are induced by radiotherapy, with a focus on metabolic sensitisation to the effects of radiotherapy. Our aim is to contribute to the development of research ideas in the field of radiotherapy metabolic-immunological studies.

## 1 Introduction

The tumor microenvironment is a complex and dynamic entity composed of various cell types and components, including tumor cells, stromal cells, extracellular matrix, and signaling molecules. Interactions among these components play crucial roles in tumor initiation, progression, recurrence, metastasis, and response to therapy (De Martino et al., 2024; Guo et al., 2023; Junttila and de Sauvage, 2013; T et al., 2025). The tumor microenvironment supports tumor growth by providing essential nutrients, shaping a favorable metabolic environment, promoting angiogenesis, and creating an immunosuppressive milieu that facilitates tumor immune evasion (Arner and Rathmell, 2023). Understanding the tumor microenvironment is essential for anti-tumor strategies, and targeting its specific components has become a prominent focus in cancer research.

Metabolic reprogramming within the tumor microenvironment supports the rapid growth and proliferation of tumor cells (Liang et al., 2024; Yang H. et al., 2024). Tumor cells preferentially utilize glycolysis for energy production even in the presence of oxygen, a phenomenon known as the Warburg effect. This metabolic shift enables tumor cells to efficiently generate ATP and biosynthetic precursors necessary for proliferation (Burns and Manda, 2017; Vander Heiden et al., 2009; Liao et al., 2024). The hypoxic conditions within the tumor further drive the reliance on glycolysis for ATP production (Altea-Manzano et al., 2025). Due to their high proliferation rate, tumor cells have increased demands for energy and biosynthetic precursors, often relying on glutamine metabolism to fulfill these requirements, maintain redox balance, and support cell survival (Keibler et al., 2016; Tufail et al., 2024; Nan et al., 2025). Additionally, tumor cells convert their lipid metabolism to support membrane synthesis and energy production, with enhanced lipogenesis and altered fatty acid oxidation being hallmark features of tumor metabolism (Koundouros and Poulogiannis, 2020; Jeon et al., 2023). These metabolic adaptations are highly plastic, allowing tumor cells to switch pathways in response to changes of nutrient and oxygen availability within the microenvironment. Furthermore, therapeutic interventions can induce metabolic stress, prompting tumor cells to modify their metabolic pathways. Inhibitors targeting tumor-associated metabolic pathways are under development as potential therapeutic approaches, which can enhance the efficacy of cancer treatments (Ricci, 2025).

Radiotherapy is a critical method for cancer treatment. High-energy radiation directly kills tumor cells by damaging their DNA and modifies the tumor microenvironment to reduce or even eliminate tumors (Zhang et al., 2022; Charpentier et al., 2022). Radiotherapy is pivotal in curative treatments, adjuvant and neoadjuvant therapies, as well as palliative care. Stereotactic body radiation therapy (SBRT), when combined with immunotherapy, introduces an innovative cancer treatment strategy that enhances the immune system’s ability to combat tumors through various mechanisms (Swamy, 2022; De Caluwe et al., 2023).

Traditionally, the effects of radiotherapy on tumors have been explained by the 5 Rs: Repair, Redistribution, Reoxygenation, Repopulation, and Radiosensitivity (Herskind et al., 2017). Recent research, however, has identified the sixth R: the Reactivation of Anti-Tumor Immune Response, which is significant in both local and systemic reactions to radiotherapy (Boustani et al., 2019). Radiotherapy not only directly kills tumor cells but also alters the tumor microenvironment, thereby reactivating the immune system to fight against tumors more effectively (Barker et al., 2015). The dual effects of radiotherapy enhance the therapeutic potential and importance in comprehensive cancer treatment strategies.

By examining the interplay between radiotherapy, metabolic alterations within the tumor microenvironment (TME), and immune responses, this review aims to provide a comprehensive understanding of how radiotherapy can enhance anti-tumor immune responses both directly and through metabolic modulation. The objective of this review is to explore the emerging research directions and develop novel therapeutic approaches in the realm of radiotherapy-induced immunity.

## 2 Metabolism in the tumor immune microenvironment (TIME)

### 2.1 Glycolysis and oxidative phosphorylation metabolism

Metabolism of tumor cells significantly differs from that of normal cells, notably in their preference for ATP production through glycolysis, even under normoxic conditions, known as the Warburg effect (Cordani et al., 2024). In tumor cells, proteins involved in oxidative metabolism are downregulated, whereas the expression of glucose and monocarboxylate transporters is upregulated. This metabolic shift enables tumor cells to uptake large quantities of glucose and rapidly generate energy via glycolysis (Kooshan et al., 2024). Additionally, increased glycolysis in tumor cells leads to elevated production of reactive oxygen species (ROS). Moderate levels of ROS can promote tumor cell proliferation and survival, while excessive ROS levels may trigger cell death (Glorieux et al., 2024; Sies et al., 2022).

**FIGURE 1:**
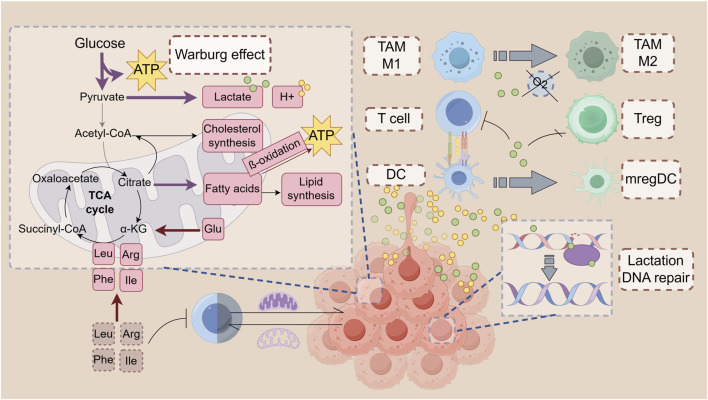
Metabolism in the tumor microenvironment: Immune modulation. Tumor cells preferentially use amino acids like arginine, glutamine, and branched-chain amino acids (BCAAs) to meet biosynthetic and energy demands. Glutamine supports cell proliferation and acts as an antioxidant, while BCAAs contribute to energy production and tumor growth. Tumor cells also use amino acid metabolism to thrive in nutrient-poor environments, with disruptions in glutamine and BCAA pathways impairing immune cell functions. CD8^+^ T Cells require arginine for survival, with its deficiency enhancing their cytotoxic activity. In contrast, CD4^+^ T Cells in low-arginine environments exhibit immunosuppressive effects. Disruptions in glutamine or leucine metabolism can impair T Cell differentiation and cytokine production, impacting immune responses. Tumor cells upregulate fatty acid synthesis and oxidation (FAO) to meet energy needs and produce signaling molecules. Fatty acids support rapid cell growth and membrane synthesis. Enzymes like fatty acid synthase (FASN) and acetyl-CoA carboxylase (ACC) are overexpressed in tumors, promoting growth and proliferation. FAO supports the energy needs of effector and memory T Cells in the tumor microenvironment (TME). However, excessive FAO can impair CD8^+^ T Cell function and promote immunosuppressive cell phenotypes in DCs and MDSCs. Cholesterol mobilization is crucial for DC maturation and effective anti-tumor immune responses. Tumor cells release unsaturated fatty acids (e.g., DHA, oleic acid) that shift TAMs to an immunosuppressive phenotype, inhibiting T Cell function and contributing to cancer progression.

Given their high proliferation rate, tumor cells require substantial energy to support rapid growth and division. Although glycolysis is less efficient at producing ATP than oxidative phosphorylation, its rapid pace provides ample energy in a short time (Cordani et al., 2024). For instance, metastatic tumor cells exhibit high glucose uptake, elevated glycolytic activity, and increased nucleic acid synthesis, supplying critical energy and materials for swift tumor growth and proliferation (Altea-Manzano et al., 2025). Low glucose levels in the tumor microenvironment can alleviate replication stress and apoptosis induced by pyrimidine synthesis inhibition by regulating metabolic diversion in tumor cells, providing a critical survival advantage and conferring resistance to pyrimidine synthesis inhibitors (Nam et al., 2024; Reinfeld et al., 2021) (Figure 1).

The accelerated glycolysis rate in tumor cells results in the accumulation of lactate and H+ ions, leading to the acidification of the extracellular environment as these metabolites are exported (Zhou et al., 2018). Subsequent imaging of the cells within the tumor indicated that myeloid cells exhibited the most significant glucose uptake, with T Cells and tumor cells following in that order (Reinfeld et al., 2021). The acidic TME facilitates tumor cell invasion and metastasis by upregulating mechanisms such as sodium-hydrogen exchanger-1 and carbonic anhydrase (Gong Y.-Y. et al., 2022; Tachibana et al., 2017). Brent A. Hanks and colleagues discovered that lactate can induce dendritic cells to adopt an immune-tolerant phenotype, transforming them into regulatory mature dendritic cells (mregDCs) (Plebanek et al., 2024). The presence of mregDCs significantly suppresses T cell-mediated anti-tumor responses, thereby aiding tumors in evading immune surveillance (Plebanek et al., 2024). Lactate can influence immune function through the form of lactylation, an epigenetic modification. The lactylation modification on histones of the macrophage genome promotes the transition of macrophages from the pro-inflammatory, anti-cancer M1 type to the anti-inflammatory, pro-cancer M2 type (Izzo and Wellen, 2019). The accumulation of lactate in the tumor microenvironment binds to and inhibits GLUT10, which impairs the glucose metabolism of CD8^+^ T Cells and suppresses the immune response (Liu Y. et al., 2024). In contrast, Tregs are less affected by the lactic acid environment. They are enriched in tumors and functionally not inhibited by metabolism; instead, they exhibit an active proliferative state, which may be one of the mechanisms by which glycolysis induces immune suppression (Watson et al., 2021; Cascone et al., 2018). Meanwhile, in a high-lactic acid environment, PD-1 inhibitors and CTLA-4 inhibitors activate PD-1+ Treg, leading to further inhibition of CD8^+^ T, which ultimately leads to treatment failure (Kumagai et al., 2022; Zappasodi et al., 2021) (Figure 1).

Lactic acid promotes cancer cell repair of broken DNA, maintains the stability of the cancer cell genome, and makes tumor cells resistant to radiation and chemotherapy (Chen et al., 2024). Acidic conditions also contribute to chemotherapy resistance by reducing the intracellular concentration of drugs like anthracyclines and vinca alkaloids *via* ion trapping (Mahoney et al., 2003). The diffusion of H+ into adjacent normal tissues can enhance glycolysis in non-tumor cells, alter tissue pH, activate metalloproteinases causing extracellular matrix degradation, increase VEGF levels, and suppress the immune response, thereby promoting tumor cell invasion and migration (Kato et al., 2013; Winkler et al., 2020; Yuan et al., 2023).

Mitochondria play a critical role in tumorigenesis, serving as indispensable organelles for ATP production and adaptation to cellular and environmental changes induced by cancer therapies. Despite the increased reliance on glycolysis, tumor cells still generate substantial energy through mitochondrial respiration. Inhibiting oxidative phosphorylation (OXPHOS) in tumor cells can impede their proliferation and enhance their sensitivity to chemotherapeutic drugs (Ma et al., 2019; Papandreou et al., 2006). Damage to mitochondria or mitochondrial DNA (mtDNA) can diminish OXPHOS efficiency, elevate ROS levels, and activate the PI3K/Akt signaling pathway, thereby promoting tumor cell proliferation (Zheng et al., 2024; Yakes and Van Houten, 1997).

Mitochondrial function of tumor-infiltrating T Cells is similarly affected by TME. CD8^+^ T Cells that are chronically and repeatedly stimulated after infiltration into a tumor secrete a cytokine called METRNL (Modulating Factor for the Regulation of Neuroglial Cell Differentiation-like Factor) due to autocrine and paracrine secretion, resulting in abnormal mitochondrial function and weakened immune response in their own right (Jackson et al., 2024). Tumor cells can extract mitochondria from natural killer cells through tunneling nanotubes (TNTs), thereby weakening the immune function of NKT cells (Saha et al., 2022; Baldwin and Gattinoni, 2022). Moreover, through the utilization of TNTs and extracellular vesicles, tumor cells can transfer mitochondria with mtDNA mutations and the USP30 protein on the mitochondrial surface into T Cells. USP30 can inhibit mitophagy, and the dysfunctional mitochondria, once replacing the normal mitochondria in T Cells, suppress T Cell activation (Ikeda et al., 2025). And delivering healthy mitochondria to T Cells enhances T Cell infiltration and prevents T Cell depletion (Baldwin et al., 2024).

Hypoxia is a critical hallmark of the TME that supports tumorigenesis and resistance to therapies. Hypoxia stabilizes hypoxia-inducible factors (HIF), preventing their ubiquitination and proteasomal degradation. HIF can upregulate membrane-bound enzymes such as carbonic anhydrase IX and XII, which help maintain an alkaline intracellular environment while acidifying the extracellular space (Lee et al., 2020). In addition to inducing acidic TME, the hypoxic environment reprograms macrophages *via* the NF-κB pathway and the methyltransferase TET2 to exhibit enhanced pro-inflammatory properties and antigen-presenting capacity, thereby enhancing their anti-tumor effects (De La Calle-Fabregat et al., 2024).

The acidic pH, accumulated lactate, and ROS in the TME can impair immune cell function. T cell-mediated immune surveillance is suppressed in an acidic environment (Kumagai et al., 2024; Pucino et al., 2019). Additionally, accumulated lactate inhibits both innate and adaptive immune responses (Manoharan et al., 2021; Sun et al., 2024). Acidic conditions lead to metabolic slowdown in T Cells and altered cytokine expression due to acidosis (Angelin et al., 2017). The acidic environment also promotes the activation of tumor-associated macrophages (TAMs), and previous research demonstrated that the acidic TME influences TAM M2 polarization through the CCL2/CCR2 axis (Bohn et al., 2018; Song et al., 2024; Yang H. et al., 2020) (Figure 1).

### 2.2 Amino acid metabolism

The metabolic preferences of tumor cells for amino acids have been the subject of extensive research. Arginine, glutamine, and branched-chain amino acids (BCAAs) are crucial for supporting the biosynthetic and energy needs of tumor cells (Wetzel et al., 2023; Bo and Fujii, 2025). Arginine deficiency in tumor cells is often linked to the loss of argininosuccinate synthetase 1 (ASS1) (Long et al., 2017). Glutamine supports nucleic acid and protein synthesis, protects cells from ROS damage, and participates in the tricarboxylic acid (TCA) cycle, making it particularly essential in rapidly proliferating tumor cells (de Oliveira et al., 2016; Shen et al., 2021). BCAAs, including leucine, isoleucine, and valine, are associated with an increased risk of colorectal adenoma and pancreatic cancer in humans (Xu et al., 2023; Katagiri et al., 2020). Tumor cells enhance their growth by upregulating BCAA metabolism, which provides intermediates for the TCA cycle (Wang et al., 2017). These metabolic adaptations allow tumor cells to thrive in nutrient-deprived and hostile microenvironments (Figure 1).

Downregulation of glutathione 1 (GPT1) expression is prevalent in colorectal tissues during the development of progressive adenomas in the normal bowel and then the malignant transformation of adenomas to colorectal cancers (CRCs) and correlates with a poor prognosis for patients (Xiong et al., 2025). It was found that GPT1 activation by the transcription factor KLF4 inhibits cancer through two mutually independent metabolic pathways, on the one hand, by increasing enzyme-dependent α-ketoglutarate (α-KG) synthesis and inhibiting the WNT signaling pathway, and on the other hand, by disrupting the folate cycle by binding to methylenetetrahydrofolate dehydrogenase (MTHFD1L) in the folate cycle (Tran et al., 2020).

CD4^+^ and CD8^+^ T Cells must maintain sufficient arginine levels to survive within the TME (Geiger et al., 2016). CD8^+^ T Cells deficient in arginase 2 (Arg2) show increased expression of perforin, granzyme, IFN-γ, and IL-2, leading to enhanced cytotoxic activity (Martí i Líndez, 2025). However, CD4^+^ T Cells are activated in an environment with low levels of arginine, and exert immunosuppressive effects (Zou et al., 2024). DCs and TAMs can synthesize arginine *via* argininosuccinate lyase (ASL) and ASS1, thereby increasing the proportion of CD4^+^ T Cells as part of T Cell regulation (Sun and Zhao, 2022). Immune cells also depend on glutamine and BCAAs to maintain homeostasis and functions properly. Deficiency in glutaminase (GLS) or leucine can impair the differentiation of Th1 and CD8^+^ T Cells and disrupt Th17 immune responses (Johnson et al., 2018). In glutamine-deprived conditions, activated CD8^+^ T Cells significantly reduce the production of cytokines such as IFN-γ and TNF-α (Nabe et al., 2018).

These insights into amino acid metabolism in tumor and immune cells highlight potential therapeutic targets and strategies for enhancing anti-tumor immunity and improving cancer treatment outcomes.

### 2.3 Fatty acid metabolism

Fatty acid metabolism, encompassing both fatty acid synthesis and oxidation, undergoes reprogramming in tumor tissues and organs. Tumor cells synthesize fatty acids to facilitate membrane biosynthesis and the production of signaling molecules (Nagarajan et al., 2021; Qin et al., 2023). Furthermore, fatty acid oxidation (FAO) supplies energy, aiding the survival of tumor cells under metabolic stress (Kant et al., 2020; Ma J. et al., 2024).

In numerous tumors, fatty acid synthesis is upregulated (Röhrig and Schulze, 2016). Tumor cells convert acetyl-CoA into fatty acids *via* a series of enzymatic reactions essential for constructing cell membranes, generating lipid signaling molecules, and producing energy (Wang H. et al., 2024; Yoshii et al., 2015). In cancer, enzymes like fatty acid synthase (FASN) and acetyl-CoA carboxylase (ACC) are often overexpressed, driving rapid cell proliferation and tumor growth. Fatty acids are also involved in synthesizing various signaling molecules (Guertin and Wellen, 2023). Arachidonic acid, a polyunsaturated fatty acid derived from linoleic acid, can be transformed through enzymatic processes into eicosanoids, such as prostaglandins, thromboxanes, and leukotrienes (Wang et al., 2021). Synthesis of glycosphingolipid, a phospholipid, is critical for successful immune escape in KRAS mutation-driven tumors: the level of glycosphingolipid synthesis correlates with the amount of interferon gamma receptor 1 (IFNGR1) on the surface of cancer cells, and blocking glycosphingolipid synthesis leads to an increase in IFNGR1, making the tumor cells more sensitive to immune responses (Soula et al., 2024). Prostaglandin E2 (PGE2) promotes tumor cell proliferation, suppresses immune cell activity, and stimulates vascular endothelial growth factor (VEGF) expression, enhancing tumor angiogenesis, nutrient, and oxygen supply, thereby supporting tumor growth and metastasis (Gong Z. et al., 2022; Terzuoli et al., 2016). PGE2 plays a critical role in suppressing the response of CD8^+^ T Cells to interleukin-2 (IL-2) signaling, thereby preventing the expansion of stem-like CD8^+^ T Cells and tumor-infiltrating CD8^+^ T Cells. This suppression ultimately leads to mitochondrial dysfunction and ferroptosis in these cells (Morotti et al., 2024; Lacher et al., 2024) (Figure 1).

FAO provides a steady energy supply for effector T Cells, enabling them to remain active within the TEM (Hunt et al., 2024). The long-term survival of memory T Cells in tumor tissues also relies on fatty acid metabolism. (Christian et al., 2021). Classical dendritic cells (cDCs) utilize their internal cholesterol reserves, derived from *de novo* synthesis and extracellular debris, to assemble lipid nanodomains on the cell surface. This process, known as “cholesterol mobilization,” upregulates the expression of maturation markers and stabilizes immune receptor signaling (Belabed et al., 2025). Cholesterol mobilization is a prerequisite for cDC maturation and subsequent antitumor immune responses (Belabed et al., 2025).

However, excessive fatty acid oxidation (FAO) can inhibit the function of CD8^+^ T Cells. In the tumor microenvironment, metabolic stress leads to increased activity of acetyl-CoA carboxylase (ACC) in T Cells, which promotes lipid storage rather than breakdown. This results in the accumulation of lipid droplets in T Cells and suppression of the FAO pathway, ultimately impairing T Cell functionality (Hunt et al., 2024). It can also induce immunosuppressive functions in DCs and myeloid-derived suppressor cells (MDSCs) (Al-Khami et al., 2017; Hossain et al., 2015; Yan et al., 2019). Inhibiting genes related to fatty acid synthesis in regulatory T (Treg) cells can enhance the anti-tumor effects of tumor-infiltrating lymphocytes (TILs) (Yang et al., 2025). Unsaturated fatty acids can modulate the phenotype of TAM, promoting their polarization to the immunosuppressive M2 phenotype (Colegio et al., 2014). In hepatocellular carcinoma (HCC), the lipid metabolism of tumor cells is altered and more long-chain unsaturated fatty acids (e.g., DHA and oleic acid) are released into the tumor microenvironment. After the unsaturated fatty acids from tumor cells are taken up by TAMs, they promote the binding of FABP5 to peroxisome proliferator-activated receptor γ (PPARγ) and enhance the transcriptional activity of PPARγ in macrophages, which may contribute to the shift of TAMs to an immunosuppressive phenotype, upregulate the expression of immunosuppressive molecules such as PD-L1, GAL1, *etc.*, and inhibit the proliferation and function of T Cells (Yang X. et al., 2024). Obesity-associated oleic acid accumulation and acid production by cancer cells leads to the accumulation of TAMs, which sense the increased acidity of the tumor microenvironment by upregulating the expression of the acid-sensing receptor GPR65, and the TAMs then downregulate their own inflammatory response, thus accelerating the occurrence and development of colorectal cancer and HCC in the obese population in the obese population (Bagchi et al., 2024).

## 3 Radiotherapy and metabolic modulation in the TIME

### 3.1 Radiotherapy effect tumor angiogenesis and immune cell infiltration

**FIGURE 2:**
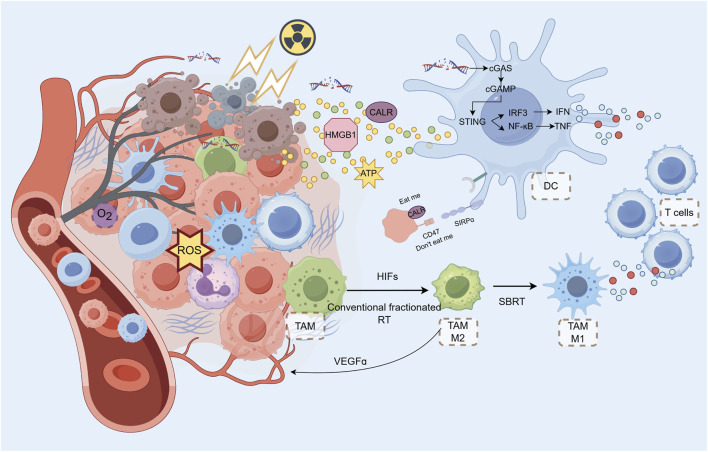
Radiotherapy and Metabolic Modulation in the TIME SBRT can cause rapid endothelial cell apoptosis and tumor vascular occlusion, leading to reduced blood supply. RT-induced cell DAMPs, activating innate immune cells and recruiting lymphocytes via chemokine release. RT induces DNA damage in tumor cells, triggering the cGAS-STING pathway. This pathway activates innate immune signaling via type I interferons (IFNs), which help initiate a systemic antitumor immune response. cGAMP, generated in response to DNA damage, activates STING, leading to the translocation of IRF3 and the production of IFNs. DAMPs like calreticulin (CALR) on tumor cells act as “eat me” signals, promoting DC-mediated phagocytosis and antigen cross-presentation. HMGB1, released during tumor cell death, binds to TLR4 on DCs to upregulate co-stimulatory molecules, enhancing T Cell activation. However, HMGB1 can also contribute to tumor metastasis, presenting a potential target for therapy. RT can increase TAM infiltration and M2 polarization, which can suppress T Cell function and promote tumor progression. RT exacerbates tumor hypoxia, which in turn activates HIFs (hypoxia-inducible factors) and promotes macrophage recruitment to the tumor. TAMs can influence tumor angiogenesis and immune suppression through factors like VEGF, TGF-β, and PD-L1 expression. While conventional fractionated RT promotes M2 polarization in TAMs, SBRT can polarize them towards the M1 phenotype, potentially enhancing the antitumor immune response. RT activates the STING pathway, promoting the antitumor functions of TANs, although their response depends on the radiation dose and fractionation schedule. High doses of radiation can transiently increase MDSC infiltration but may also activate TANs, enhancing tumor regression through ROS production.

Despite the lack of evidence from human studies, studies using syngeneic or xenograft tumor models have shown that low doses (<5 Gy) of radiotherapy can cause short-term vascular damage to tumor tissue (Castle and Kirsch, 2019; Clement et al., 1978). In contrast, blood supply to tumor tissue is consistently reduced after irradiation above 5 Gy or 10 Gy. High doses of radiation (≥10 Gy) delivered in SBRT can trigger rapid apoptosis of vascular endothelial cells within 1–6 h, causing tumor vascular occlusion and secondary tumor-killing effects (Castle and Kirsch, 2019). Irradiation of Walker 256 carcinoma tumors in rats showed that irradiation above 10 Gy caused a sustained reduction in blood flow to the tumor tissue (Song et al., 1972) (Figure 2).

It is intuitive that tumor vascular occlusion is deleterious to immune cell infiltration. However, evidence indicates that conventional fractionated radiotherapy, hyperfractionated radiotherapy, and hypofractionated radiotherapy can, in fact, promote tumor-localized immune cell infiltration (Guo et al., 2023; Cytlak et al., 2022).

Irradiation of a portion of the tumor while sparing the peritumoral immune environment has been demonstrated to transform the immune-suppressive to immune-stimulatory effect, thereby enhancing immune-mediated anti-tumor effects (Tubin et al., 2020). Studies have shown a significant increase in the total number of lymphocytes within TME after SBRT (van der Woude et al., 2022; Wang J. et al., 2024). The effect of SBRT on immune cell infiltration remains controversial, and infiltrating immune cells are not always beneficial for tumor therapy. Mills et al (Mills et al., 2022) conducted an analysis of the changes in the immune microenvironment of human pancreatic ductal adenocarcinoma following SBRT. SBRT alone resulted in a reduction in tumor cell density while an increase in immunogenic cell death. SBRT promoted collagen deposition; however, the vascular system remained unaltered, and spatial analysis provided no evidence of T Cell segregation (Mills et al., 2022). In contrast, The proportion of PD-1 (+) T Cells increased after SBRT (Mills et al., 2022). SBRT resulted in a reduction of tertiary lymphoid structures and failed to reduce or reprogram abundant immunosuppressive cell populations. Tumor-associated macrophages (TAMs) suppress collagen biosynthesis by initiating a transforming growth factor-β-driven collagen biosynthesis program (Tharp et al., 2024; Maller et al., 2021). Collagen synthesis depletes the microenvironment of arginine and further drives proline synthesis and ornithine secretion, making the metabolic microenvironment less favorable for CD8^+^ T Cells, thereby suppressing the anti-tumor T Cell immune response (Tharp et al., 2024; Maller et al., 2021). de Perrot et al (de Perrot et al., 2020) also found that radiotherapy increased PD-L1 positivity and increased the proportion of TILs in mesothelioma tissue (Figure 2).

The increased immune cell infiltration induced after SBRT can be explained by several mechanisms. First, the effect of RT on angiogenesis may not be isolated: Filipa Gil Marques et al. found that low-dose fractionated irradiation (0.8 Gy/f) activated peritumoral endothelial cells (Gil Marques et al., 2020). Microvessel density (MVD) was also significantly increased in peritumoral tissues treated with low-dose fractionated irradiation (Gil Marques et al., 2020). At the same time, there is a window of vascular normalization for the anti-angiogenic effect of RT, where there is a cycle of generation and destruction of intra-tumoral vasculature, and immune cells can still reach the inside of the tumor through normalized vessels (Jain, 2005).

Radiotherapy-induced cell death can result in the release of DAMPs, which can activate innate immune cells *via* the pattern recognition receptor (PRR), leading to the release of chemokines. These chemokines can recruit circulating immune cells to the tumor site (Cytlak et al., 2022). RT can cause activation of macrophages and DCs by inducing cell surface translocation of calreticulin, extracellular release of high-mobility group protein B1 (HMGB1), and release of adenosine triphosphate (ATP) (Krysko et al., 2012). Activated innate immune cells recruit lymphocytes to the tumor site by releasing chemokines such as CXCL9, CXCL10, and CXCL16, as well as IL-1ß, TNFɑ, and type I and type II interferons. Irradiated vascular endothelial cells simultaneously increase the expression of intercellular adhesion molecule (ICAM)-1 and vascular cell adhesion molecule (VCAM)-1, thereby increasing the adhesion of circulating immune cells (Hallahan et al., 1996).

### 3.2 cGAS-STING signal pathway

The cGAS-STING signaling pathway plays a crucial role in modulating both metabolic and immune responses in tumors following radiotherapy (Jiang et al., 2025; Nakajima et al., 2023; Jesenko et al., 2020).

Radiation therapy directly kills cells by two mechanisms: direct DNA damage from energetic particles and the indirect effects of ROS formation (Eriksson and Stigbrand, 2010; Kim et al., 2019). Both modes of killing have also become sources of immunogenic DNA. Tumor cell nuclear DNA and mitochondrial genome (mtDNA) that is damaged beyond repair produces dsDNA (Vodicka et al., 2025). cGAS in tumor cells forms a 2:2 DNA oligomerization complex with dsDNA. cGAS acts as a catalyst to promote the synthesis of the second messenger 2′3′-looped GMP-AMP (cGAMP) and activates STING in tumor cells (Zhao et al., 2025). dsDNA can also be ingested into the extracellular compartment by other tumor cells. The extracellularly released dsDNA can also be taken up by other tumor cells or by immune cells to synthesize cGAMP (Diamond et al., 2018). cGAMP promotes the translocation of STING to the Golgi to activate TANK-binding kinase 1 (TBK1), which phosphorylates STING and promotes the translocation of interferon regulatory factor 3 (IRF3) to the nucleus, promoting the production of type I IFNs and triggering subsequent innate immune signaling (Hopfner and Hornung, 2020). In addition to DNA, cytoplasmic tumor-derived cGAMP can also act as a second messenger that can diffuse through gap junctions to neighboring cells (Ma X. et al., 2024). This intercellular and extracellular signaling underlies the systemic antitumor effects of RT.

The NF-κB signaling pathway is primarily activated through a STING-TBK1-dependent mechanism. There are two pathways: the classical and the alternative (Sun, 2017). In the classical pathway, activated STING phosphorylates TBK1, which in turn stimulates IκB kinase (IKK) (Lin et al., 2023; Yum et al., 2021). IKK phosphorylates the NF-κB inhibitor protein IκB, leading to its ubiquitination by the proteasome (Cohen and Strickson, 2017). Following IκB degradation, NF-κB is released to form the p50/RelA dimer (Muxel et al., 2016). This dimer translocates to the nucleus to trigger the transcription of type I interferons and pro-inflammatory factors (Deng et al., 2014). However, the alternative NF-κB pathway (p100/RelB) is also activated (Keogh et al., 2017). The alternative NF-κB pathway is involved in regulating the development of primary and secondary lymphoid organs, the survival and maturation of B Cells, as well as the maturation and function of DCs (Sun, 2017).

The ionizing radiation used in radiotherapy interacts with molecules in cells to generate ROS, including superoxide, nitrogen oxides, hydroxyl radicals, and others (Bao et al., 2024; Pietraforte et al., 2018). In addition to DNA, these ROS can also cause oxidative damage to lipids. Lipid peroxidation refers to the process in which ROS react with polyunsaturated fatty acids (PUFAs) that are abundant in cell membranes (Pearson et al., 2021). Excessive lipid peroxidation can lead to cell ferroptosis, which is characterized by the accumulation of lipid peroxides (Lei et al., 2020). The activation of cGAS can enhance the process of lipid peroxidation in cells, promoting cell ferroptosis (Liu H. et al., 2024). Furthermore, the type I interferons and pro-inflammatory cytokines produced after the activation of the cGAS-STING signaling pathway can exacerbate tissue damage (Li et al., 2023).

### 3.3 Metabolic-immune microenvironment altered by radiotherapy

#### 3.3.1 Dendritic cells (DCs)

DCs are regulated in a variety of ways in the tumor microenvironment following radiotherapy. In addition to the release of large amounts of tumor antigens, radiotherapy also releases related substances that play a role in the modulation of innate immunity (Del Prete et al., 2023). These include inflammation-related cytokines such as IFNs, IL-1, IL-6, IL-8, VEGF, EGFR, and TNFα (Kany et al., 2019; Kopf et al., 2010). These cytokines create a localized inflammatory environment in the early phase of radiotherapy (Kopf et al., 2010). Moreover, DAMPs such as calreticulin surface translocation and HMGB1 and ATP released by cell death can be recognized by PRRs such as TLRs on the surface and inside the DC, triggering an immune response (Ashrafizadeh et al., 2020; Apetoh et al., 2007). In addition, radiotherapy-induced DNA double-strand break can both act as DAMP molecules and activate the STING (interferon gene stimulating factor) pathway to promote type I interferon production (Cytlak et al., 2022). Activation of this pathway helps to initiate a robust innate and adaptive immune response, supporting the efficacy of immunotherapy.

HMGB1 is released from dead cells. By binding to TLR4 on the surface of DCs, HMGB1 can upregulate the co-stimulatory molecules CD80 and CD86, contributing to the subsequent activation of T Cells. Radiation can cause immunogenic death of tumor cells by inducing the release of HMGB1 (Saenz et al., 2014; Yamazaki et al., 2014). However, HMGB1 released by tumor vesicles can also promote tumor invasion and metastasis through a series of post-transcriptional regulations (He et al., 2018). By blocking HMGB1 and the TLR4 receptor on immune cells, solid tumor growth can be inhibited (Wen et al., 2018; Chen et al., 2019). Hubert et al (Hubert et al., 2021) achieved some results using HMGB1 inhibitors in combination with anti-PD-L1 antibodies in the treatment of mouse breast cancer. Therefore, HMGB1 may become a regulatory target for anti-tumor therapy (Figure 2).

Radiotherapy can increase the expression of CALR on tumor cells, which acts as a pro-phagocytic “eat me” signal for DCs, facilitating their recognition and engulfment of tumor cells (Luo et al., 2023). High expression of calreticulin promotes intra-tumor infiltration of DC and CD4^+^ Th1 cells. However, CD47, an anti-phagocytic “do not eat me” signal, is often highly expressed on tumor cells, counteracting the effects of CALR. Blocking CD47 with monoclonal antibodies promotes phagocytosis of tumor cells mediated by DCs, which in turn enhances antigen cross-presentation and activation of cytotoxic T Cells (Hsieh et al., 2022). The combination of radiotherapy and CD47 blockade has been shown to be synergistic in reducing tumor growth and improving survival in animal models (Nishiga et al., 2022). Furthermore, the overexpression of CALR induced by the combination of a Jak inhibitor (ruxolitinib) and CD47 blockade (magrolimab) in myelofibrosis cells suggests a potentiated pro-phagocytic signal that could enhance the antitumor immune response (Boasman and Simmonds, 2023). In essence, the balance between the pro-phagocytic signal CALR and the anti-phagocytic signal CD47 on tumor cells is critical for the ability of DCs to recognize and phagocytose tumor cells, determining effective antigen cross-presentation and activation of a robust antitumor immune response during radiotherapy (Preet Kaur et al., 2023). Co-expression of mitochondrial FAO enzymes (CPT1A, CPT2, and ACAD9) and the immune checkpoint CD47 is predominant in patients with recurrent GBM with poor prognosis (Jiang et al., 2022). Metabolic reprogramming of glycolysis to FAO was associated with CD47 anti-phagocytosis in radioresistant GBM cells and with synthesis of GBM regrowth after radiation in mice (Jiang et al., 2022).

The cGAS-STING signaling pathway induces type I interferon expression, in which the classical and non-classical NF-ĸB pathways play important roles (Zhang and Zhang, 2025). cGAS-STING signaling after RT also activates the NF-ĸB pathway. The classical NF-ĸB pathway and IRF3 effectively promote the secretion of type I IFN in DCs stimulated by irradiated tumor cells (Yu et al., 2020). However, RT-induced IFN release of DCs could be inhibited by activation of the non-classical NF-ĸB pathway (Iwanaszko and Kimmel, 2015). Activation of STING is also regulated by DNases. Radiotherapy above 12 Gy upregulates the expression of 3′-repair exonuclease 1 (TREX1), degrades dsDNA and thus reduces activation of the cGAS/type I IFN pathway (Li et al., 2024). TLR9 agonists have been demonstrated to augment the number of DCs, CD4^+^, and CD8^+^ T Cells following radiotherapy, thereby enhancing the local and systemic anti-tumor effects of RT (Younes et al., 2021).

Similar mechanisms have been validated in clinical patients. Increased expression of tumor-associated antigens in tumor tissue following radiation therapy (Singh et al., 2017). High levels of Fas ligand (FASLG) and galectin 1 (Gal-1) were associated with clinical benefit. Targeted sequencing of tissue DNA and circulating tumor DNA (ctDNA) showed a high degree of genomic concordance (Sundahl et al., 2019). At the same time, these immune-related proteins were not elevated in matched patients receiving chemotherapy (Christensen et al., 2024).

These antigens are taken by DCs to induce following immune responses. DCs radiation-irradiated in the tumor are found in the tumor-draining lymph nodes (TdLN) as migratory CD103+ cDC1 and CD11b+ cDC2 subsets. In a subset of tumors that are insensitive to radiotherapy, the low percentage of conversion to mature CD103+ DCs may be a mechanism for poor treatment response (Blair et al., 2022). Tumors upregulate type I IFN after radiation therapy, which is dependent on STING expression in host cells, indicating a host source of type I IFN transcripts. The authors demonstrated that macrophages and DCs are the major source of type I IFN transcripts (Deng et al., 2014).

#### 3.3.2 Tumor-associated macrophages (TAMs)

TAMs have been the subject of numerous studies and have significant implications for tumor development and therapy (Vitale et al., 2019; Rodriguez-Ruiz et al., 2020). The majority of studies have classified these cells into two main polarization types: the M1 type, which has pro-inflammatory effects, and the M2 type, which has inhibitory effects (Cassetta and Pollard, 2020). RT also increased the number of TAMs and the expression of stromal cell-derived factor-1 (SDF-1) and HIF-1 at the front of tumor invasion (Wang et al., 2013) (Figure 2).

Radiotherapy-induced vascular injury serves to exacerbate the hypoxic environment of the tumor, leading to the upregulation of HIF and other hypoxic factors (Maeda et al., 2017). This, in turn, results in the chemotaxis of macrophages to the tumor site *via* the CXCR4 pathway (Ceradini et al., 2004). Hypoxia-inducible factors (HIFs) represent a class of key molecules in the cellular response to hypoxia. In normal oxidic conditions, HIF-α is continuously synthesised and subjected to ubiquitination, which leads to its degradation by the proteasome (Kietzmann et al., 2016). In hypoxic conditions, HIF-a is less ubiquitinated and enters the nucleus to participate in the hypoxic response (Zhang et al., 2025). Furthermore, CCL2 is dependent on the induced expression of HIF1a (Baay-Guzman et al., 2012). In TAMs, HIF-1a and HIF-2a accumulate in the nucleus during hypoxia, promoting CSF1R and CXCR4 expression (Kozin et al., 2010; Chiang et al., 2012). TAMs have been observed to modulate HIF-1a expression in tumor cells and the tumor endothelium subsequent to low-dose radiotherapy. In addition, iNOS + TAM cells have been shown to activate eNOS + endothelial cells, thereby promoting angiogenesis (Nadella et al., 2018). High expression of Arg-1, COX-2, and iNOS after routinely fractionated radiotherapy with TAMs has also been found in a mouse model of prostate cancer to promote early tumor growth (Tsai et al., 2007). The TAMs are also known to be highly regulated by the TAMs themselves. Hypoxic TAMs strongly upregulate the expression of the mTOR negative regulator REDD1 (He and Zhang, 2021). REDD1-mediated mTOR inhibition impedes glycolysis in TAMs and interferes with the pro-angiogenic response of TAMs, leading to the formation of abnormal blood vessels (Wenes et al., 2016). In a mouse model of glioma, RT decreased the MVD in the core of primary tumors but increased the MVD in the tumor infiltration front (Wang et al., 2013).

These macrophages arriving at the tumor site tend to be M2 polarised and play an inhibitory role in the tumor immune microenvironment. The direct release of DAMPs, such as ROS, and the activation of the NF-κB pathway by radiotherapy appear to promote the polarization of TAMs towards the M2 phenotype (Guo et al., 2024). The binding of the activated p50-p50 dimer to NF-kB resulted in M2-polarised TAMs expressing elevated levels of IL10 and TGF-b, which in turn inhibited the activation of downstream immune pathways (Crittenden et al., 2012). Additionally, the high expression of PD-L1 on M2 TAMs led to the death of CD8 T Cells and the suppression of T Cell function. Interestingly, in contrast to conventional fractionation, which promotes polarization of TAMs towards M2, radiotherapy with hypofraction polarized TAMs towards M1, suggesting another potential advantage of SBRT (Becherini et al., 2023) (Figure 2).

#### 3.3.3 Tumor-associated neutrophils (TANs) and myeloid-derived suppressor cells (MDSCs)

Neutrophils represent a crucial innate immune cell within the tumor microenvironment. Radiotherapy has been demonstrated to release type I interferon through the activation of the STING pathway. This pathway has been shown to promote anti-tumor functions of TANs, including the production of ROS, MMP-8, and FasL (Lu S. et al., 2024; Lu D. et al., 2024). The effects of radiotherapy on TAN function are contingent upon the dosage and segmentation mode employed (Luo et al., 2024; M et al., 2024).

MDSCs represent a significant cellular component of the tumor microenvironment (Li et al., 2021). This class of cells has its origin in various progenitor cells of the bone marrow and exerts a suppressive effect on immune function. Conventional fractionated radiotherapy resulted in the accumulation of MDSCs in the tumor site, rendering the tumor resistant to radiotherapy (Kho et al., 2021). Radiotherapy at 8 Gy × 3 F induced phosphorylation of histone H2AX (γH2AX), leading to the secretion of CXCL1, CXCL2, and CCL5 inflammatory chemokines (Hu et al., 2023; Liu et al., 2021; Olive and Banáth, 2004). A high-dose of radiation (26 Gy) rapidly and transiently infiltrates tumors with CD11b+Gr-1high + neutrophils (Liu et al., 2014). Neutrophils recruited by radiation exhibit increased ROS production and induced apoptosis in tumor cells. In the final analysis, tumor-specific cytotoxic T Cells are activated, recruited to the tumor site, and cause tumor regression. The combined administration of granulocyte colony-stimulating factor (G-CSF) has been demonstrated to enhance the antitumor activity of radiotherapy by activating TANs (Takeshima et al., 2016).

The secretion of Arg1 by MDSCs results in the deprivation of the tumor microenvironment of L-arginine, which in turn leads to the downregulation of the TCR-ζ chain (Yang Y. et al., 2020; Feng et al., 2024). Additionally, MDSCs exhibit overexpression of indoleamine 2,3-dioxygenase 1 (IDO1), which is associated with an increased infiltration of Tregs in tumors and metastatic lymph nodes (Holmgaard et al., 2015; Wang et al., 2025). Polymorphonuclear (PMN)-MDSCs function through expressing NADPH oxidase and producing ROS, whereas monocyte (M)-MDSCs express inducible nitric oxide synthase (iNOS) and produce nitric oxide (NO) (Herring et al., 2022; Palmieri et al., 2020). The presence of elevated levels of ROS has been validated to induce apoptosis in T Cells and result in the downregulation of the TCR ζ chain (Chen et al., 2016). Upon reaction with NO, ROS nitrosylates the TCR, resulting in a lack of T Cell responsiveness to antigen (Belikov et al., 2014). MDSCs secrete immunosuppressive cytokines such as TGF-β and IL-10, which reduce the antitumor activity of effector T Cells (Yang Y. et al., 2020). Furthermore, MDSCs inhibit T Cell migration to peripheral lymph nodes by reducing the expression of CD62L on the surface of immature T Cells, thereby inhibiting T Cell activation (Ferrer et al., 2021; Peng et al., 2021). Activation of T Cells A series of cytokines produced by tumor cells, including TGF-β, IL-6, IL-10, and GM-CSF, can induce MDSCs to produce ROS, which facilitates the expression of FasL and inhibits the expression of Bcl2, thereby inducing apoptosis of activated T Cells (Yadav et al., 2023; Dong, 2021). Additionally, MDSCs play an immunosuppressive role by upregulating PD-L1, which binds to the PD-1 receptor on T Cells, leading to programmed cell death (Liu W. et al., 2024).

## 4 Therapeutic approaches combining radiotherapy and immune metabolism modulation

The combination of radiotherapy with metabolic-immune modulation therapy is still being explored. Primary research interests include deregulating RT and tumor metabolism induced immunosuppression TIME and influencing RT sensitivity by modulating metabolism.

### 4.1 Reducing immune suppression caused by RT and metabolism

The activation of cytotoxic T-lymphocyte-associated antigen 4 (CTLA-4), programmed cell death protein 1 (PD-1), and programmed death-ligand 1 (PD-L1) impairs the ability of T Cells to activate, recognize, and eliminate cancer cells, thereby bypassing anti-tumor immune surveillance and consequently diminishing the therapeutic efficacy of RT (Zhang et al., 2021).

Currently, several clinical trials have combined ICIs with RT to treat solid tumors. Examples include the START-FIT trial for unresectable hepatocellular carcinoma (HCC) (Chiang et al., 2023), the CHEERS trial for various metastatic tumors (Spaas et al., 2023; Spaas et al., 2021), and the FORCE trial for non-small cell lung cancer (NSCLC) (Bozorgmehr et al., 2018; Bozorgmehr et al., 2020a), the PACIFIC trial (Antonia et al., 2018), and the PEMBRO-RT trial (Theelen et al., 2019). There are also ongoing studies such as TRADE-hypo trial (Bozorgmehr et al., 2020b; Bozorgmehr et al., 2021), the NeoCheckRay trial (De Caluwe et al., 2023; De Caluwé et al., 2021), and the NIRVANA-Lung trial (Doyen et al., 2022). These studies have explored the therapeutic efficacy of combinations of different ICIs and different RT regimens in a wide range of tumors (Pitroda et al., 2009).

### 4.2 Regulating metabolism to enhance radiosensitivity and ICI efficiency

A research indicates that metformin, a well-known anti-diabetic drug, may also have anti-tumor effects. Metformin has been shown to reduce hypoxia in TME by reducing the expression of HIF-1 and angiogenesis-related factors, thereby enhancing the anticancer effects of programmed cell death protein 1 (PD-1) inhibitors (Scharping et al., 2017). Immune failure is a common phenomenon after ICI treatment. Metformin enhances CD8 activity by restoring the function of depleted cytotoxic T Cells and protecting them from apoptosis (Cha et al., 2018). Metformin also decreases the stability and membrane localization of programmed death ligand 1 (PD-L1) through an AMPK-dependent mechanism, resulting in reduced PD-L1 expression (Cha et al., 2018). Metformin inhibits lung cancer cell growth and enhances their sensitivity to ionizing radiation (IR) by modulating the ATM-AMPK signaling pathway. Moreover, metformin has been shown to increase the radiosensitivity of esophageal cancer, pancreatic cancer, and colorectal cancer cells *in vitro*, of which effects are linked to AMPK activation, indicating that AMPK is crucial in regulating radio-sensitizing effects of metformin (Storozhuk et al., 2013).

Drugs targeting the glutamine and arginine metabolic pathways are also of interest. CB-839 (telaglenastat) is an oral glutaminase inhibitor that blocks tumor consumption of glutamine. This blockade increases glutamine levels in TIME and enhances immune cell activity (Meric-Bernstam et al., 2016). Agnello et al (Agnello et al., 2020) investigated pegzilarginase, a PEGylated arginine-degrading enzyme, demonstrating its safety and antitumor efficacy in preclinical settings. The authors found that cancers with low expression of argininosuccinate synthase (ASS1), such as malignant melanoma and small cell lung cancer, were particularly sensitive to pegzilarginase treatment. Importantly, the study highlighted the immune-potentiating effects of arginine depletion, which synergizes with T-cell-mediated immunity. However, the study’s limitation lies in its reliance on preclinical models, necessitating further clinical validation.

Similarly, Leone et al (Leone et al., 2019) explored the role of glutamine metabolism in tumor immune evasion. By using a glutamine antagonist, they revealed divergent metabolic responses between cancer cells and effector T Cells, showing that glutamine blockade suppresses tumor metabolism while enhancing T Cell oxidative metabolism and activation. This “metabolic checkpoint” strategy provides a novel avenue for immunotherapy. Furthermore, Tannir et al (Tannir et al., 2022) conducted a randomized clinical trial (CANTATA) to evaluate telaglenastat, a glutaminase inhibitor, in combination with cabozantinib for advanced renal cell carcinoma (RCC). Despite promising preclinical synergy, the trial found no significant improvement in progression-free survival with telaglenastat compared to placebo. While the combination was well-tolerated, the lack of efficacy suggests challenges in translating preclinical metabolic interventions into clinical success. A potential limitation of this study is the heterogeneity of prior treatments among patients, which may have influenced outcomes.

In head and neck squamous cell carcinoma (HNSCC), studies have found that the tumor immune microenvironment and tumor metabolism are key determinants of chemoradiotherapy response. Immunohistochemical analysis of pre-treatment tumor biopsies from 73 HNSCC patients receiving radical chemoradiotherapy revealed that mitochondrial-rich (COX5B) metabolism correlated with higher intra-tumoral CD8/CD4 ratios, whereas glucose-dependent (GLUT1) metabolism correlated with lower ratios (Krupar et al., 2018). A high CD8/CD4 ratio, coupled with mitochondrial-rich or glucose-independent metabolism, was associated with improved short term survival. Prognostic analysis of CD8A, GLUT1, and COX5B gene expression in The Cancer Genome Atlas (TCGA) database further confirmed that patients with favorable immune and metabolic gene characteristics (high CD8A, high COX5B, low GLUT1) had better short and long term survival. Additionally, *in vitro* experiments showed that the glycolysis inhibitor 2-deoxyglucose and radiotherapy synergistically upregulated the chemotactic effect of ROS-dependent peripheral blood mononuclear cells (PBMCs). (Krupar et al., 2018).

Shao et al (Shao et al., 2024) designed an innovative therapeutic nano-platform to induce cuproptosis and synergize with low-dose radiotherapy (LDRT, 0.5–2 Gy) for treating *in situ* HCC. The approach reversed the hypoxic tumor microenvironment, promoting a metabolic shift from glycolysis to OXPHOS, thereby enhancing sensitivity to cuproptosis. Simultaneously, the Fenton-like reaction ensured a continuous copper supply and glutathione (GSH) depletion, disrupting mitochondrial function and interrupting energy supply (Shao et al., 2024).

## 5 Challenges and future directions

Radiotherapy impacts metabolism and the TIME through multiple mechanisms, influencing immune responses. Numerous clinical studies combining radiotherapy and immunotherapy have confirmed improved patient outcomes. However, the immunosuppressive effects of radiotherapy and the TME itself limit therapeutic efficacy. Future research should focus on mitigating the adverse effects of radiotherapy on metabolism and immunity to enhance its role in cancer treatment. Current studies suggest that combining radiotherapy with targeted metabolic pathway therapies has significant potential for development.

Given the profound influence of the TIME on cancer treatment, there is a need for convenient and reliable biomarkers that can sensitively and specifically reflect metabolic changes within the TIME. Personalized metabolic biomarker detection will support the development of individualized treatment plans, reducing the adverse effects of radiotherapy and immunotherapy. The development of novel biomarkers, in combination with radiotherapy and immunotherapy, can provide better prognostic guidance for patients.

Additionally, integrating radiotherapy with metabolic-immune regulation to enhance the synergistic effects of radiotherapy and immunotherapy requires the collaborative efforts of radiation oncologists, immunologists, and metabolism experts.

## 6 Summary

In summary, metabolic alterations in the TIME significantly shape immune responses, influencing tumor progression, immune evasion, and the effectiveness of therapies. Targeting these metabolic pathways holds promise for improving cancer treatment outcomes by enhancing immune function and overcoming the immunosuppressive effects of the TIME.

Radiotherapy induces tumor cell death, releasing DAMPs that activate innate immune responses, particularly through the STING pathway, leading to increased type I interferon production. This activation affects various immune cells, including DCs, TAMs, TANs, and MDSCs. While radiotherapy can promote immune infiltration, it also induces immune suppression in the tumor microenvironment, with TAMs typically polarizing to an M2 phenotype and MDSCs dampening T Cell activity. The combination of radiotherapy with metabolic modulation, such as the use of metformin or targeting metabolic shifts (e.g., from glycolysis to oxidative phosphorylation), holds promise for enhancing the therapeutic response. The challenges of the tumor’s immunosuppressive microenvironment highlight the need for personalized therapies, with biomarkers to guide treatment decisions and optimize patient outcomes.
